# Idiopathic male infertility is strongly associated with aberrant DNA methylation of imprinted loci in sperm: a case-control study

**DOI:** 10.1186/s13148-018-0568-y

**Published:** 2018-10-29

**Authors:** Qiuqin Tang, Feng Pan, Jing Yang, Ziqiang Fu, Yiwen Lu, Xian Wu, Xiumei Han, Minjian Chen, Chuncheng Lu, Yankai Xia, Xinru Wang, Wei Wu

**Affiliations:** 1 Department of Obstetrics, The Affiliated Obstetrics and Gynecology Hospital of Nanjing Medical University, Nanjing Maternity and Child Health Care Hospital, Nanjing, China; 2 Department of Urology, The Affiliated Obstetrics and Gynecology Hospital of Nanjing Medical University, Nanjing Maternity and Child Health Care Hospital, Nanjing, China; 3 State Key Laboratory of Reproductive Medicine, Institute of Toxicology, Nanjing Medical University, 101 Longmian Avenue, Nanjing, 211166 China; 4 Key Laboratory of Modern Toxicology of Ministry of Education, School of Public Health, Nanjing Medical University, Nanjing, China; 5 National Toxicology Program Laboratory, Division of the National Toxicology Program, National Institute of Environmental Health Sciences, Research Triangle Park, NC USA; 6 Department of Health and Human Services, National Institute of Environmental Health Sciences, National Institutes of Health, Research Triangle Park, USA

**Keywords:** DNA methylation, DNMT, Global methylation, Imprinted gene, Male infertility, Polymorphism, Sperm

## Abstract

**Background:**

Male infertility is a complex disease caused by a combination of genetic, environmental, and lifestyle factors. Abnormal epigenetic programming has been proposed as a possible mechanism compromising male fertility. Recent studies suggest that aberrant imprinting in spermatozoa in a subset of infertile men is a risk factor for congenital diseases in children conceived via assisted reproduction techniques. In this study, we examined the DNA methylation status of CpG sites within the differentially methylated regions (DMRs) of three imprinted genes, *H19*, *GNAS*, and *DIRAS3*, using combined bisulfite PCR restriction analysis and bisulfite sequencing in sperm obtained from 135 men with idiopathic male infertility, including normozoospermia (*n* = 39), moderate oligozoospermia (*n* = 45), and severe oligozoospermia (*n* = 51), and fertile controls (*n* = 59). The percentage of global methylation was compared between fertile controls and infertile patients displaying abnormal DNA methylation status of imprinted loci. Moreover, we also analyzed whether the DNA methyltransferases (DNMTs) polymorphisms impact upon the methylation patterns of imprinted genes in idiopathic infertile males.

**Results:**

Aberrant methylation patterns of imprinted genes were more prevalent in idiopathic infertile males, especially in patients with oligozoospermia. Infertile males with aberrant methylation patterns of imprinted genes displayed a tendency of lower global methylation levels, although not reaching statistical significance (*P* = 0.13). In the genotype-epigenotype correlation analysis, no significant association was observed between aberrant methylation patterns of the three imprinted genes and genotypes of the four DNA methyltransferase (DNMT) genes.

**Conclusion:**

We conclude that abnormalities of DMR within imprinted genes may be associated with idiopathic male infertility. Disruption in methylation pattern of the three imprinted genes does not occur in high-risk genotypes of DNMTs.

**Electronic supplementary material:**

The online version of this article (10.1186/s13148-018-0568-y) contains supplementary material, which is available to authorized users.

## Introduction

Male infertility is a multifactorial disorder which affects approximately 15% of couples at reproductive age globally with substantial clinical and social impact [1]. In spite of the magnitude of the problem and the considerable research efforts that have been made to understand its causes, a large proportion of male infertility cases remains idiopathic in nature [2]. In these patients, oligozoospermia is frequently observed, and most infertile men resort to some kinds of assisted reproductive technique (ART). Even though this method allows the infertile males to father their own children without knowing the cause of their infertility, it also carries the potential risk of transmitting genetic or epigenetic defects and impacting offspring [3, 4].

Genomic imprinting is an epigenetic phenomenon which mediates mono-allelic, parent-of-origin-specific expression of genes during embryonic development and after birth [5, 6]. Genomic imprints are erased in primordial germ cells and are newly established during later stages of germ cell development, which are stably inherited through somatic cell divisions during postzygotic development [7]. DNA methylation at differentially methylated regions (DMRs) is one of the regulatory mechanisms controlling the allele-specific expression of imprinted genes. DNA methylation of CpG dinucleotides at DMRs is the most studied epigenetic mark [7–10]. Genomic imprinting is vital for normal gene expression patterns in an individual, with errors sometimes resulted in inappropriate gene transcription or repression [6]. Aberrant regulation of imprinted genes is known to be responsible for various growth and behavioral syndromes [11]. Because imprinted genes escape epigenetic reprogramming after fertilization and maintain their parent-specific germline patterns, aberrant methylation imprints can be transmitted directly from the father’s sperm into the developing embryo [12].

It has been suggested that intracytoplasmic sperm injection (ICSI), by canceling the natural selection of aberrant spermatozoa, may cause irregular embryo cell divisions due to the mechanical manipulation of the gametes and might transmit genetically related infertility [13, 14]. Although the overall rate of congenital anomalies in children conceived by ART is low (4–6%), this rate still represents a significant increase compared with the background rate of major malformations (3%) [15]. Several clinical studies reported an unexpected high incidence of imprinting disorders (e.g., Angelman syndrome (AS) and Beckwith-Wiedemann syndrome (BWS)) in children conceived with ART [16, 17]. Additionally, the demonstration of imprinting defects in cases of disrupted spermatogenesis raised the possibility that they could be associated with infertility itself [18]. Several studies have shown that infertile males with impaired spermatogenesis are prone to have a higher incidence of aberrant methylation in imprinted genes [18–26].

Although hints exist that spermatozoa from the infertile male can carry imprinting defects, relatively small sample size patient cohorts were investigated and few clinical details were given. In addition, most studies of the methylation status of imprinted genes usually select men diagnosed as infertile without the inclusion of any appropriate fertile controls [18–23]. These results can directly demonstrate the potential risk of imprinting defect on semen quality, but not male infertility. In this study, to test our hypothesis that imprinting defect is associated with idiopathic male infertility, we utilized carefully the selected fertile controls to evaluate the association between the imprinting defect of three imprinted genes and idiopathic male infertility. In addition, we analyzed the global methylation level of sperm DNA which represents epigenetic changes affecting multiple loci and explored the association between genotypes of four DNA methyltransferase (DNMT) polymorphisms and risk of aberrant methylation of imprinted genes.

## Methods

### Subject recruitment and sample collection

Study subjects were volunteers form affiliated hospitals of Nanjing Medical University between July 2009 and September 2010. The study was approved by the Institutional Review Board of Nanjing Medical University, and subjects gave written informed consent. All activities involving human subjects were done under full compliance with the government policies and the Helsinki Declaration. Consecutive eligible men (without diagnosed infertile wives) were recruited to participate, 196 in total were asked. Of those approached, 87.2% consented (171 participants). A completed physical examination including height and weight was performed, and a questionnaire was used to collect information including personal background, lifestyle factors, environmental and occupational exposures, genetic risk factors, and medical history. Men with immune infertility, semen non-liquefaction, and medical history of risk factors for infertility (e.g., varicocele, orchidopexy, or postvasectomy), and receiving treatment for infertility (e.g., hormonal treatments) were excluded from the study (15 of 171 subjects). Men with other known causes related to male infertility, such as genetic disease, infection, and occupational exposure to the agents suspected to be associated with male reproduction, were also excluded (13 of 156 subjects). Furthermore, to avoid azoospermia or severe oligozoospermia caused by Y chromosome microdeletions, we excluded subjects with Y chromosome microdeletions of the azoospermia factor region (8 of 143 subjects). We selected 59 fertile men from the early pregnancy registry, from the same hospitals as the cases. All controls were healthy men with normal reproductive function and confirmed to have healthy babies 6–8 months later. All subjects were Han Chinese who came from Nanjing and neighborly suburban area.

### Semen analysis

Semen samples were obtained in private by masturbation into a sterile wide-mouth and metal-free glass container after a recommended at least 3-day sexual abstinence. After liquefaction at 37 °C for 30 min, conventional semen analysis was conducted in accordance with the guidelines of the WHO Laboratory Manual for the Examination of Human Semen (World Health Organization, 1999), including semen volume, sperm number per ejaculum, sperm concentration, motility, progression, and motion parameters. Strict quality control measures were enforced throughout the study. Observation and counting in the semen analysis were automatic, and the backgrounds of the samples were blinded to avoid bias.

### DNA extraction

Motile sperm cells were purified away from lymphocyte contamination, immature germ cells, and epithelial cells using a Percoll (GE Healthcare) gradient with two concentration layers (80% and 40%). A microscopic examination of the sperm fractions was performed to control the quality of cell preparations. The sperm pellets were resuspended twice in phosphate-buffered saline (PBS) and centrifuged at 400*g* for 10 min. Genomic sperm DNA isolation was performed as described [27]. DNA concentration was determined by spectrophotometry.

### Bisulfite treatment and methylation analysis

**Fig. 1:**
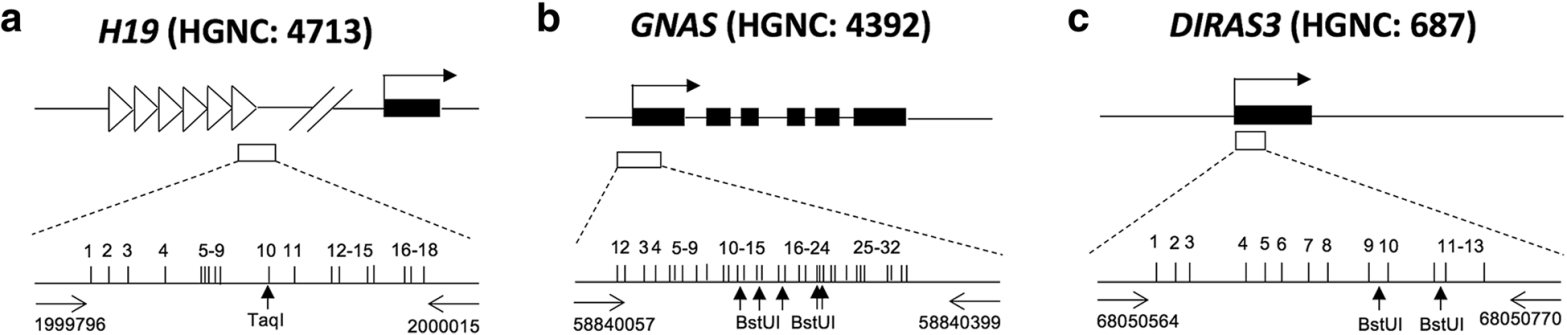
Structural characteristics of the human DMRs of *H19* (**a**), *GNAS* (**b**), and *DIRAS3* (**c**). Filled boxes and horizontal arrows indicate the genes and orientation, respectively. Open boxes represent the DMRs of the genes. The horizontal arrows represent the primers. Vertical arrows indicate the unique bisulfite PCR restriction enzyme sites analyzed in T, *Taq*I, and B, *Bst*UI. The vertical bars represent CpG sites

Methylation assays were performed at the DMRs of three imprinted genes [*H19* (HGNC: 4713), *GNAS* (HGNC: 4392), and *DIRAS3* (HGNC: 687)] in humans using combined bisulfite PCR restriction analysis (COBRA) and bisulfite sequencing PCR (BSP). Genomic DNA (1 μg, or 500 ng when sperm DNA was not enough) was treated with sodium bisulfite using the EpiTect Bisulfite Kit (Qiagen) according to the protocol recommended by the manufacturer. Bisulfite converts unmethylated cytosine to uracil while 5-methylcytosine (5-MeC) remains unchanged. Three microliters of the final eluent was used for subsequent PCR amplification. PCR included an initial incubation at 94 °C for 5 min, followed by 40 cycles of 94 °C for 40 s; 60 °C (for *H19* and *GNAS*) and 54 °C (for *DIRAS3*) for 40 s; and 72 °C for 60 s, followed by one cycle of 72 °C for 10 min. The amplification products were separated on 2% agarose gels and stained with ethidium bromide. Most samples have been subject to two independent bisulfite treatments and were analyzed from two independent PCR products. The region analyzed for each of these genes was within the DMR of CpG islands. We examined 18 CpG sites in a 220-bp fragment of *H19* (chromosome 11: 1999796-2000015), 32 CpG sites in a 343-bp fragment of *GNAS* (chromosome 20: 58840057-58840399), and 13 CpG sites in a 207-bp fragment of *DIRAS3* (chromosome 1: 68050564-68050770) (Fig. 1).

To ensure that the sequencing results did not reflect a cloning bias, restriction analysis carried out on germ cell and somatic cell DNA, cutting the DNA with enzymes that could cleave only the methylated templates of the same bisulfite-treated PCR samples. After amplification, 20–50% of PCR products were digested with the restriction enzyme *TaqI* (New England Biolabs) for *H19* and *BstUI* (New England Biolabs) for *GNAS* and *DIRAS3*, both of which recognize sequences unique to the methylated (bisulfite-unconverted) templates but cannot recognize unmethylated (bisulfite-converted) templates. DNA was then electrophoresed on a 3% agarose gel. The intensity of methylated templates was calculated by densitometry using AlphaEaseFC software (Alpha Innotech), and the percentage of methylated restriction enzyme site in each genomic sample was calculated from the ratio between the enzyme-cleaved PCR products and the total amount of PCR products. In addition, the PCR products were purified and cloned into the pCR2.1 vector by TA Cloning kit (Invitrogen, Carlsbad, CA, USA). To determine the methylation status of DMR, an average of ten clones (each plate) were sequenced using M13 reverse primer and an automated ABI Prism 3730xl Genetic Analyzer (Applied Biosystems, Foster City, CA, USA).

### Global methylation analyses

For each sample, methylation analysis was performed in triplicate and averaged (100 ng DNA each) using a Methylamp Global DNA Methylation Quantification Kit (Epigenetek, New York, NY) following the manufacturer’s instructions. Briefly, the methylated fractions of DNA were recognized by an anti-5-methylcytosine antibody and quantified through an enzyme-linked immunosorbent assay-like reaction. The total amount of methylated DNA is proportional to the optical density (OD). Positive control and the negative control were provided with the kit and included in each experiment as an internal control. Global methylation percentage values were normalized to this internal control.

### Variant genotyping

Genotype analyses of four polymorphisms in *DNMT1*, *DNMT3A*, *DNMT3B*, and *DNMT3L* were performed using the DNA obtained from the patient’s blood. Genotypes were detected by allelic discrimination assay using TaqMan® MGB probes on a 7900HT Fast real-time PCR system (Applied Biosystems, CA). The gene variants studied were *DNMT1* (rs4804490), *DNMT3A* (rs1550117), *DNMT3B* (rs2424909), and *DNMT3L* (rs7354779) that have been described in our previous study [28]. Very rare variants are of limited value in association studies as they are only relevant to a small proportion of the population and their effects are difficult to detect in practice. Therefore, we selected only variants where the frequency of the minor allele was > 5%. For the quality control, the genotyping was done without the knowledge of case/control status of the subjects, a random 10% of cases and controls were genotyped twice by different individuals, and the reproducibility was 100%.

### Statistical analysis

Analysis of variance was used to explore the relationships between fertility status and potentially important covariates, such as age, body mass index (BMI), ejaculate volume, sperm concentration, and motility. The *χ*^2^ test was used to evaluate the differences in smoking status, alcohol drinking status, duration of sexual abstinence, and the frequency of aberrant methylation of imprinted genes between case and control groups. Logistic regression analysis was performed to obtain the odds ratios (ORs) for idiopathic male infertility and aberrant methylation and 95% confidence intervals (95% CI) with adjustment for age, BMI, smoking status, alcohol drinking, and duration of sexual abstinence, wherever it was appropriate. Results of global methylation are expressed as mean ± SD. All statistical analyses were carried out using Stata (Version 9.0, StataCorp, LP), and *P* ≤ 0.05 was considered to be significant.

## Results

We first analyzed the DNA methylation pattern of the three imprinted genes in sperm DNA samples from 135 idiopathic infertile males and 59 fertile male controls. Demographic categories by fertility and semen quality are described in Table 1. Briefly, each group of cases and controls were well matched for age, BMI, smoking status, and alcohol drinking (*P* > 0.05). As the analyzed genes were chosen as indicators of paternal and maternal imprinting, > 90% methylation was expected for the paternally imprinted *H19* and < 10% for the maternally imprinted *DIRAS3* and *GNAS* in sperm DNA [20].

**Table 1:** Characteristics of idiopathic infertile males and fertile controls

Characteristic	Controls (*n* = 59)^a^	Cases			
		Case 1 (*n* = 39)^b^	Case 2 (*n* = 45)^c^	Case 3 (*n* = 51)^d^	Case all (*n* = 135)^e^
Age (years, mean ± SD)	29.08 ± 3.85	29.85 ± 4.84	28.71 ± 3.91	28.78 ± 4.07	29.07 ± 4.25
BMI (kg/m^2^, mean ± SD)^f^	23.93 ± 4.17	22.61 ± 3.54	22.99 ± 2.72	22.92 ± 3.36	22.86 ± 3.20
Smoking status [*n* (%)]					
No	27 (45.8)	21 (53.8)	26 (57.8)	23 (45.1)	70 (51.9)
Yes	32 (54.2)	18 (46.2)	19 (42.2)	28 (54.9)	65 (48.1)
Alcohol drinking [*n* (%)]					
No	46 (78.0)	33 (84.6)	37 (82.2)	41 (80.4)	111 (82.2)
Yes	13 (22.0)	6 (15.4)	8 (17.8)	10 (19.6)	24 (17.8)
Abstinence time [*n* (%)]					
< 4	15 (25.4)	7 (18.3)	10 (23.8)	4 (12.1)	21 (15.6)
4–7	38 (64.4)	41 (68.3)	24 (57.1)	23 (69.7)	88 (65.2)
≥ 7	6 (10.2)	12 (13.3)^g^	8 (19.0)	6 (18.2)	26 (19.3)
Ejaculate volume (ml, mean ± SD)	4.29 ± 1.32	2.99 ± 1.21^g^	3.30 ± 1.60^g^	3.43 ± 1.47^g^	3.26 ± 1.45^g^
Sperm concentration (10^6^/ml, mean ± SD)	49.52 ± 33.85	76.92 ± 54.36^g^	14.62 ± 2.91^g^	4.35 ± 2.75^g^	28.74 ± 42.58^g^
Sperm motility (%, mean ± SD)	57.00 ± 16.05	53.81 ± 26.54	39.59 ± 20.12^g^	31.48 ± 14.70^g^	40.64 ± 22.27^g^

^a^Control: fertile men

^b^Case 1: idiopathic infertile men with normozoospermia (sperm concentration ≥ 20 × 10^6^/ml)

^c^Case 2: idiopathic infertile men with moderate oligozoospermia (sperm concentration 5–20 × 10^6^/ml)

^d^Case 3: idiopathic infertile men with severe oligozoospermia (sperm concentration < 5 × 10^6^/ml)

^e^Case all: the sum of case 1, case 2, and case 3

^f^*BMI* body mass index

^g^*P* < 0.05 when compared between case and control groups

**Fig. 2:**
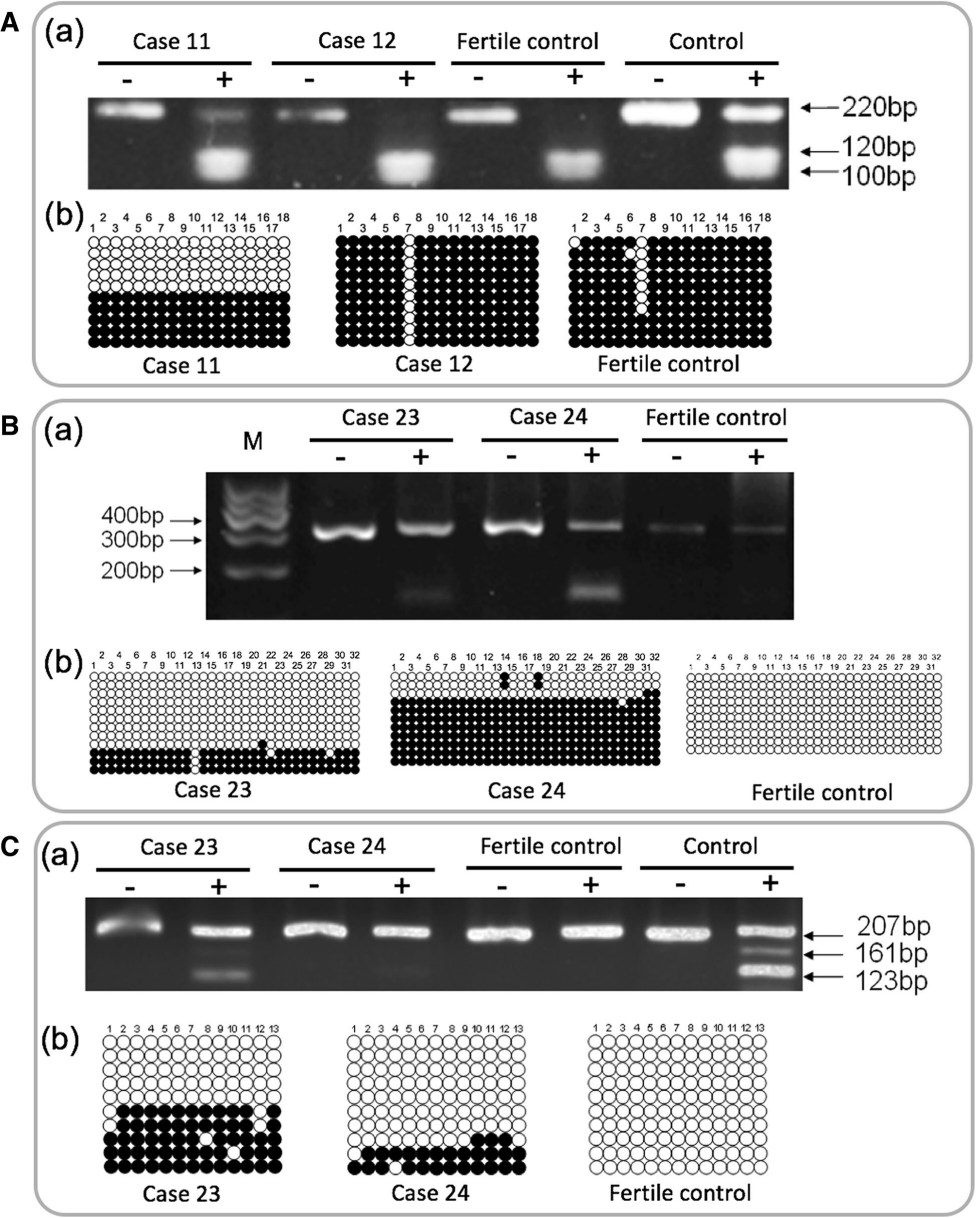
Examination of methylation patterns of the three imprinted genes in the sperm of infertile males and fertile controls by combined bisulfite PCR restriction analysis (COBRA) and bisulfite-sequencing PCR (BSP). **A** Representative results of the COBRA (a) and BSP (b) analysis of *H19*. **B** Representative results of the COBRA (a) and BSP (b) analysis of *GNAS*. **C** Representative results of the COBRA (a) and BSP (b) analysis of *DIRAS3*. Filled and open circles represent methylated and unmethylated CpGs, respectively. M, molecular weight markers; Control, blood DNA (hemimethylated DNA)

In total, we identified the aberrant methylation of *H19*, *GNAS*, and *DIRAS3* in 19.3%, 21.5%, and 22.2% of the 135 infertile males, respectively (Additional file 1: Table S1). The frequencies of aberrant methylation of the two maternal imprinted genes (*GNAS* and *DIRAS3*) were higher than those of the paternal imprinted gene (*H19*). However, cases with severe oligozoospermia were more prone to have aberrant methylation of the paternal imprinted gene than maternal imprinted genes (Additional file 1: Table S1). Notably, among 26 cases with aberrant methylation of *H19*, 29 cases with aberrant methylation of *GNAS*, and 30 cases with aberrant methylation of *DIRAS3*, 6 had all the three imprinted genes abnormally methylated, 9 had both *H19* and *GNAS* abnormally methylated, 8 had both *H19* and *DIRAS3* abnormally methylated, and 11 had both *GNAS* and *DIRAS3* abnormally methylated. Aberrant methylation of the three imprinted genes was also found in fertile controls (Table 2). Adjusted ORs and 95% CIs for associations between idiopathic male infertility and aberrant methylation status of imprinted genes are presented in Table 2. Compared with men of normal methylation status of imprinted genes, men with aberrant methylation status were more likely to have idiopathic infertility (for *H19*, OR = 7.61, 95% CI = 1.71–33.80; for *GNAS*, OR = 17.53, 95% CI = 2.29–134.19; for *DIRAS3*, OR = 9.02, 95% CI = 2.01–40.58; Table 2; Fig. 2), especially patients with severe oligozoospermia (for *H19*, OR = 28.52, 95% CI = 5.85–139.06; for *GNAS*, OR = 25.35, 95% CI = 3.10–207.00; for *DIRAS3*, OR = 19.79, 95% CI = 3.69–106.03; Table 2; Fig. 1). For a more detailed analysis, the subjects were categorized according to the concentration (< 5, 5–20, ≥ 20 million sperm/ml) of sperm. In all the three subgroups, there was a statistically significant increased frequency of aberrant methylation of *GNAS*. In contrast, the statistically significant increased frequency of aberrant methylation was observed only in case 3 subgroup for *H19* and in case 2 and case 3 subgroups for *DIRAS3* (Table 2). In addition, most patients with abnormalities in both imprint regions were severe oligozoospermia. These results suggested that aberrant methylation of imprinted genes was associated with increased idiopathic male infertility risks, while the idiopathic infertile subjects with oligozoospermia (especially severe oligozoospermia) might be at higher risk.

**Table 2:** Adjusted ORs (95% CIs) for idiopathic male infertility by methylation status of imprinted genes

Gene	Methylation	Control (*n* = 59)^a^	Case							
			Case 1 (*n* = 39)^b^		Case 2 (*n* = 45)^c^		Case 3 (*n* = 51)^d^		Case all (*n* = 135)^e^	
		*n* (%)	*n* (%)	OR (95% CI)^f^	*n* (%)	OR (95% CI)^e^	*n* (%)	OR (95% CI)^e^	*n* (%)	OR (95% CI)^e^
H19	Normal	57 (96.6)	37 (94.9)	1.00	44 (97.8)	1.00	28 (54.9)	1.00	109 (80.7)	1.00
	Aberrant	2 (3.4)	2 (5.1)	1.66 (0.20–13.48)	1 (2.2)	0.60 (0.05–7.43)	23 (45.1)	28.52 (5.85–139.06)	26 (19.3)	7.61 (1.71–33.80)
GNAS	Normal	58 (98.3)	33 (84.6)	1.00	36 (80.0)	1.00	37 (72.5)	1.00	106 (78.5)	1.00
	Aberrant	1 (1.7)	6 (15.4)	12.89 (1.35–122.79)	9 (20.0)	14.91 (1.76–126.58)	14 (27.5)	25.35 (3.10–207.00)	29 (21.5)	17.53 (2.29–134.19)
DIRAS3	Normal	57 (96.6)	35 (89.7)	1.00	35 (77.8)	1.00	35 (68.6)	1.00	105 (77.8)	1.00
	Aberrant	2 (3.4)	4 (10.3)	4.17 (0.61–28.58)	10 (22.2)	9.11 (1.76–47.16)	16 (31.4)	19.79 (3.69–106.03)	30 (22.2)	9.02 (2.01–40.58)

ORs adjusted for age, BMI, smoking status, alcohol drinking, and abstinence time

^a^Control: fertile men.

^b^Case 1: idiopathic infertile men with normozoospermia (sperm concentration ≥ 20 × 10^6^/ml)

^c^Case 2: idiopathic infertile men with moderate oligozoospermia (sperm concentration 5–20 × 10^6^/ml)

^d^Case 3: idiopathic infertile men with severe oligozoospermia (sperm concentration < 5 × 10^6^/ml)

^e^Case all: the sum of case 1, case 2, and case 3

**Fig. 3:**
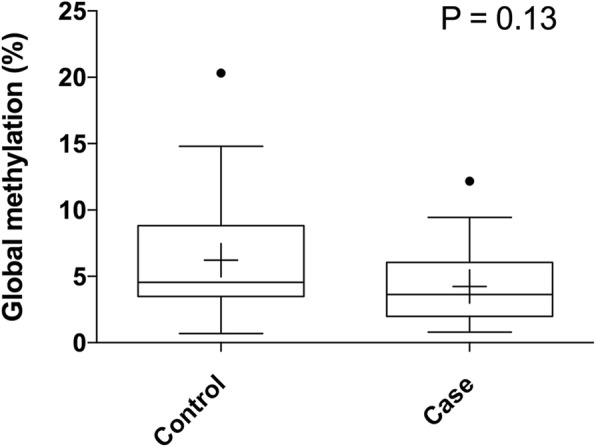
Box plots of global DNA methylation in sperm. Methylation levels of infertile males and fertile controls are given in Tukey box plots showing median (−) and mean (+) values

To further investigate whether sperm samples from patients with aberrant methylation of imprinted genes displayed global DNA methylation changes, we analyzed the global methylation of infertile men with aberrant methylation of imprinted genes (*n* = 20) and fertile controls (*n* = 20). Infertile men displayed a tendency of lower global methylation levels (4.25 ± 2.99% in infertile men versus 6.21 ± 4.83% in fertile controls), although not reaching statistical significance (*P* = 0.13; Fig. 3).

The previous study suggested that the epigenetic causal variation of male infertility should be considered together with genetic variations [29]. Therefore, we sought to analyze the correlation between the polymorphisms of DNMTs and aberrant methylation of imprinted genes in the sperm of idiopathic infertile males. The genotypes of the four polymorphisms in DNMTs (rs4804490 in *DNMT1*, rs1550117 in *DNMT3A*, rs2424909 in *DNMT3B*, and rs7354779 in *DNMT3L*) were analyzed in the blood DNA of patients. Six patients showed aberrant methylation of all the three imprinted genes, four with wild-type homozygous of all four DNMTs, one with heterozygous of rs4804490, and one with variant homozygous of rs4804490. However, no statistically significant associations were observed between the distribution of any of the DNMT polymorphisms and aberrant methylation patterns of any of the imprinted genes as shown in Additional file 2: Table S2.

## Discussion

This study adds to the growing literature that defects in spermatogenesis could be associated with the epigenetic regulation of imprinting in the male germ line [14, 30]. The DNA methylation of imprinted genes is established during spermatogenesis and maintained in mature spermatozoa. In fertile men with a normal ejaculate, paternal DMRs of the DNA should be methylated and the maternal DMRs should be unmethylated. Interestingly, even some fertile controls exhibited aberrant methylation of the three imprinted genes. As recently reported that environmental and lifestyle risk factors may affect DNA methylation of the imprinted gene in human sperm [10, 31], we speculated that the aberrant methylation of these fertile men might be caused by environmental and lifestyle risk factors. Future studies are required to address this problem. In idiopathic infertile males, however, it showed variable aberrant methylation patterns that were proportional to the severity of the oligozoospermic phenotype. In this study, loss of methylation was detected in the paternally imprinted and methylated DMRs of *H19*, whereas the maternally imprinted and unmethylated DMR of *GNAS* and *DIRAS3* showed increased methylation in idiopathic infertile males, suggesting a simultaneous hypo- and hypermethylation of the haploid spermatozoa genome. Abnormal methylation was detected at the DMR of *H19* in 26 of 135 patients (19.3%), and one case of complete absence of methylation was observed. However, the patients with *H19* completely unmethylated did not acquire aberrant methylation of *GNAS* or *DIRAS3*.

In idiopathic infertile males, DNA methylation in sperm is frequently affected at one or multiple imprinting control regions. The studied loci represent only a small fraction of imprinted genes. However, methylation abnormalities in these imprinted genes may be considered as indicators for more profound epimutation at other loci. Therefore, we compared the global methylation levels of sperm DNA in idiopathic infertile males with aberrant methylation of imprinted genes and fertile controls. Alterations in tumor DNA methylation include locus-specific hypermethylation and generalized genome-wide hypomethylation [32, 33]. No significant difference in global methylation was observed between idiopathic infertile males and fertile controls but displayed a tendency of lower global methylation levels. Global hypomethylation is associated with genomic instability and an increased number of mutational events [34–37]. Furthermore, we found that the group with oligozoospermia had a higher frequent aberrant methylation of imprinted genes compared with the group with normozoospermia, suggesting a role of DNA methylation in regulating spermatogenesis in human males. We speculated that the altered methylation patterns of imprinted genes could be due to an improper erasure and establish of methylation imprints in the early germ cells since both gains, and loss of DNA methylation were observed and global methylation seemed unaffected.

Reduced sperm counts, low progressive motility, and abnormal morphology are indications for ART. We considered that low birth weight [38], congenital malformations [39, 40], and imprinting disorders [41] in some ART children can, at least partially, be attributed to epigenetic variations. The association between ART and both AS and BWS was studied and confirmed by research groups from the US, Europe, and Australia [16, 17, 42]. A previous study suggested that the increase in the incidence of imprinting disorders in individuals born by ART may be attributed to the use of sperm with imprinting defects [14]. The aberrant patterns of methylation at DMRs observed in sperm of oligozoospermic men could be transmitted to the zygote and might affect the expression of imprinted genes and phenotype in the developing embryo.

It has been noted that the developmental outcome of ART is generally poor when sperm showed to have an abnormal DNA methylation pattern [43]. Importantly, a previous study demonstrated that aberrant DNA methylation of DMRs in children of oligozoospermia males was inherited from the father [14]. Therefore, it is necessary to add imprint methylation analysis to the routine sperm examination to identify preexisting imprint mutations when ART is applied in the fertility clinic to aid idiopathic infertile males to become fathers. Future studies are needed to determine whether infertility will be transmitted to offspring conceived by in vitro fertilization using the sperm of infertile men with aberrant methylation of imprinted genes. Additionally, we should pay special attention to the contamination of somatic cells in the purified sperm sample. Though we have done our very best to purify sperm and include a microscope to control the quality of cell preparations, contamination may remain a problem.

DNA methylation is performed by a group of proteins termed DNMTs. Embryogenesis is severely impaired in Dnmt1- or Dnmt3b-knockout mice, and spermatogenesis is impaired in Dnmt3a-knockout mice [44, 45]. DNMT3L, which is similar to DNMT3, is unique among DNMTs because it lacks enzymatic activity. The consequence of Dnmt3L deficiency in mice is the loss of imprints [46]. Our recent study has shown that DNA sequence variants were more prevalent in patients with oligozoospermia [28]. Therefore, we also analyzed the association between genetic variations of four DNMTs and the risk of aberrant methylation of imprinted genes. However, no significant association between the distribution of polymorphisms of DNMTs and risk of aberrant methylation of any of the imprinted genes was observed.

The findings obtained in the present study suggest that aberrant methylation patterns of imprinted genes were associated with the risk of spermatogenic failure in the Chinese population, although the global methylation levels do not seem to be associated with male infertility. Our results strengthen the premise that abnormal epigenetic reprogramming of the male germ line is a possible mechanism of compromised idiopathic male infertility. Our findings support the urgent need for research to better understand the potential mechanisms for aberrant methylation of imprinted genes in human spermatogenesis and the related pathology.

## Additional files

Additional file 1: **Table S1.** Proportions of aberrant methylation at three imprinted genes in sperm samples. (DOCX 14 kb)

Additional file 2: **Table S2.** Genotype frequencies of *DNMTs* in patients with normal and aberrant methylation of the three imprinted genes and their association with methylation patterns. (DOCX 20 kb)

## Abbreviations

5-MeC: 5-Methylcytosine
ART: Assisted reproductive technique
AS: Angelman syndrome
BMI: Body mass index
BSP: Bisulfite sequencing PCR
BWS: Beckwith-Wiedemann syndrome
COBRA: Combined bisulfite PCR restriction analysis
DMRs: Differentially methylated regions
DNMTs: DNA methyltransferases
ICSI: Intracytoplasmic sperm injection
OD: Optical density
PBS: Phosphate-buffered saline
