# Supplementary material for: Idiopathic male infertility is strongly associated with aberrant DNA methylation of imprinted loci in sperm: a case-control study
Source: Clin Epigenetics. 2018 Oct 29;10:134. doi: 10.1186/s13148-018-0568-y (PMC6206675; doi:10.1186/s13148-018-0568-y)
Supplement: Supplementary file 1 — Table S1. Proportions of aberrant methylation at three imprinted genes in sperm samples. (DOCX 14 kb) [file 13148_2018_568_MOESM1_ESM.docx]

**Table S1.** Proportions of aberrant methylation at three imprinted genes in sperm samples.

| **Group** | **Abnormal methylation** | | |
| --- | --- | --- | --- |
|  | **H19** | **GNAS** | **DIRAS3** |
|  | **n (%)** | **n (%)** | **n (%)** |
| **Fertile controls** | 2/59 (3.4) | 1/59 (1.7) | 2/59 (3.4) |
| **Infertile males** | 26/135 (19.3)^*^ | 29/135 (21.5)^*^ | 30/135 (22.2)^*^ |
| Normalzoospermia | 2/39 (5.1) | 6/39 (15.4)^*^ | 4/39 (10.3) |
| Moderate oligozoospermia | 1/45 (2.2) | 9/45 (20.0)^*^ | 10/45 (22.2)_*_ |
| Severe oligozoospermia | 23/51 (45.1)^*^ | 14/51 (27.5)^*^ | 16/51 (31.4)^*^ |

^*^ *P* < 0.05.
