# Supplementary material for: Idiopathic male infertility is strongly associated with aberrant DNA methylation of imprinted loci in sperm: a case-control study
Source: Clin Epigenetics. 2018 Oct 29;10:134. doi: 10.1186/s13148-018-0568-y (PMC6206675; doi:10.1186/s13148-018-0568-y)
Supplement: Supplementary file 2 — Table S2. Genotype frequencies of DNMTs in patients with normal and aberrant methylation of the three imprinted genes and their association with methylation patterns. (DOCX 20 kb) [file 13148_2018_568_MOESM2_ESM.docx]

**Table S2.** Genotype frequencies of *DNMTs* in patients with normal and aberrant methylation of the three imprinted genes and their association with methylation patterns.

| **Gene (SNPs)** | **Genotype** | **H19** | | | **GNAS** | | | **DIRAS3** | | |
| --- | --- | --- | --- | --- | --- | --- | --- | --- | --- | --- |
|  |  | **Normal methylation** | **Abnormal methylation** | | **Normal methylation** | **Abnormal methylation** | | **Normal methylation** | **Abnormal methylation** | |
|  |  | **n (%)** | **n (%)** | **OR (95% CI)^a^** | **n (%)** | **n (%)** | **OR (95% CI)^a^** | **n (%)** | **n (%)** | **OR (95% CI)^a^** |
| DNMT1 (rs4804490) | CC | 28 (27.7) | 10 (38.5) | 1.00 | 29 (29.6) | 9 (31.0) | 1.00 | 28 (28.6) | 10 (34.5) | 1.00 |
|  | CA | 51 (50.5) | 11 (42.3) | 0.61 (0.22-1.66) | 48 (49.0) | 14 (48.3) | 0.99 (0.37-2.67) | 47 (48.0) | 15 (51.7) | 0.99 (0.38-2.61) |
|  | AA | 22 (21.8) | 5 (19.2) | 0.62 (0.17-2.24) | 21 (21.4) | 6 (20.7) | 0.95 (0.28-3.21) | 23 (23.5) | 4 (13.8) | 0.47 (0.12-1.91) |
|  | A-allele carriers | 73 (72.3) | 16 (61.5) | 0.57 (0.22-1.45) | 69 (70.4) | 20 (69.0) | 0.97 (0.38-2.43) | 70 (71.4) | 19 (65.5) | 0.82 (0.33-2.06) |
| DNMT3A (rs1550117) | GG | 71 (67.6) | 20 (76.9) | 1.00 | 68 (66.7) | 23 (79.3) | 1.00 | 71 (69.6) | 20 (69.0) | 1.00 |
|  | GA | 32 (30.5) | 6 (23.1) | 0.77 (0.27-2.19) | 32 (31.4) | 6 (20.7) | 0.52 (0.18-1.47) | 30 (29.4) | 8 (27.6) | 0.86 (0.32-2.34) |
|  | AA | 2 (1.9) | 0 (0.0) | - | 2 (2.0) | 0 (0.0) | - | 1 (1.0) | 1 (3.4) | 5.17 (0.24-111.89) |
|  | A-allele carriers | 34 (32.4) | 6 (23.1) | 0.73 (0.26-2.08) | 34 (33.3) | 6 (20.7) | 0.49 (0.17-1.38) | 31 (30.4) | 9 (31.0) | 0.97 (0.37-2.54) |
| DNMT3B (rs2424909) | CC | 85 (84.2) | 22 (84.6) | 1.00 | 80 (81.6) | 27 (93.1) | 1.00 | 81 (81.8) | 26 (92.9) | 1.00 |
|  | CT | 16 (15.8) | 4 (15.4) | 1.10 (0.31-3.83) | 18 (18.4) | 2 (6.9) | 0.27 (0.05-1.39) | 18 (18.2) | 2 (7.1) | 0.25 (0.05-1.30) |
|  | TT | 0 (0.0) | 0 (0.0) | - | 0 (0.0) | 0 (0.0) | - | 0 (0.0) | 0 (0.0) | - |
|  | T-allele carriers | 16 (15.8) | 4 (15.4) | 1.10 (0.31-3.83) | 18 (18.4) | 2 (6.9) | 0.27 (0.05-1.39) | 18 (18.2) | 2 (7.1) | 0.25 (0.05-1.30) |
| DNMT3L (rs7354779) | TT | 83 (94.3) | 22 (95.7) | 1.00 | 80 (94.1) | 25 (96.2) | 1.00 | 77 (92.8) | 28 (100) | 1.00 |
|  | TC | 4 (4.5) | 1 (4.3) | 0.93 (0.09-9.88) | 4 (4.7) | 1 (3.8) | 0.59 (0.06-6.19) | 5 (6.0) | 0 (0.0) | - |
|  | CC | 1 (1.1) | 0 (0.0) | - | 1 (1.2) | 0 (0.0) | - | 1 (1.2) | 0 (0.0) | - |
|  | C-allele carriers | 5 (5.7) | 1 (4.3) | 0.93 (0.09-9.79) | 5 (5.9) | 1 (3.8) | 0.49 (0.05-5.30) | 6 (7.2) | 0 (0.0) | - |

^a^ORs adjusted for age, BMI, smoking status, alcohol drinking, and abstinence time.
